# What do grid cells contribute to place cell firing?

**DOI:** 10.1016/j.tins.2013.12.003

**Published:** 2014-03

**Authors:** Daniel Bush, Caswell Barry, Neil Burgess

**Affiliations:** 1 University College London (UCL) Institute of Cognitive Neuroscience, London, WC1N 3AR, UK; 2 UCL Institute of Neurology, London, WC1N 3BG, UK; 3 UCL Department of Cell and Developmental Biology, London, WC1E 6BT, UK

**Keywords:** place cells, grid cells, boundary vector cells, border cells, hippocampus

## Abstract

The unitary firing fields of hippocampal place cells are commonly assumed to be generated by input from entorhinal grid cell modules with differing spatial scales. Here, we review recent research that brings this assumption into doubt. Instead, we propose that place cell spatial firing patterns are determined by environmental sensory inputs, including those representing the distance and direction to environmental boundaries, while grid cells provide a complementary self-motion related input that contributes to maintaining place cell firing. In this view, grid and place cell firing patterns are not successive stages of a processing hierarchy, but complementary and interacting representations that work in combination to support the reliable coding of large-scale space.

## Highlights

• It is commonly assumed that grid cell inputs generate hippocampal place fields, but recent empirical evidence brings this assumption into doubt.
• We suggest that place fields are primarily determined by environmental sensory inputs.
• Grid cells provide a complementary path integration input and large-scale spatial metric.
• Place and grid cell representations interact to support accurate coding of large-scale space.

## Spatially modulated firing in the hippocampal formation

**Figure 1:**
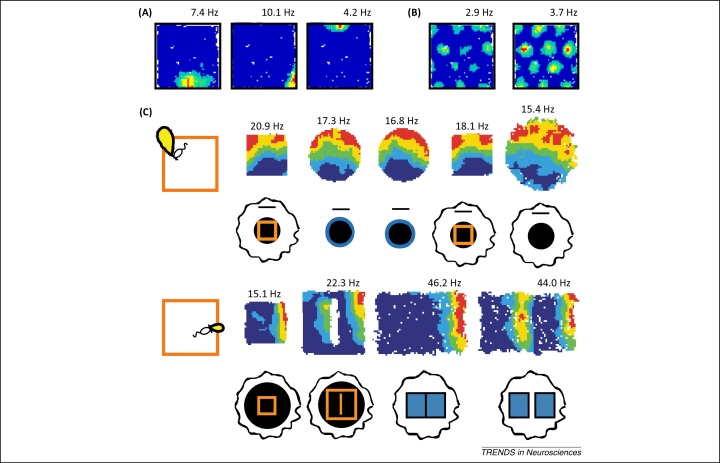
Spatially modulated firing in the hippocampal formation. **(A)** Firing rate maps for three simultaneously recorded CA1 place cells (adapted from [68]). **(B)** Firing rate maps for two simultaneously recorded grid cells in dorsal medial entorhinal cortex (mEC) (adapted from [68]). **(C)** Putative tuning curves (left panel) and firing rate maps for two subicular boundary cells recorded in multiple environments, illustrating the constant relationship between their firing fields and local borders within each environment (adapted from [10]). Superscript indicates peak firing rate.

The medial temporal lobes, and hippocampus in particular, have long been implicated in episodic and spatial memory function in humans and animals respectively 1, 2, 3. Early *in vivo* electrophysiology studies, seeking to identify the behavioural or cognitive correlates of neural activity in this region, established that the firing of principal cells in rodent hippocampus is primarily determined by the location of the animal [4]. These ‘place cells’ are typically active in a single area within a given environment – the corresponding ‘place field’ (Figure 1A) – and have been hypothesised to support a cognitive map of known locations in rodents, and episodic memory in humans [3]. Decades of subsequent research have attempted to establish the sensory stimuli and neural mechanisms that support their rapidly expressed, highly specific and spatially stable firing patterns. During this time, several other spatially responsive cell types have been identified in the hippocampal formation (Box 1). The next to be discovered were head direction cells, which encode the head direction of the animal in the horizontal plane independently of location 5, 6, 7. More recently, grid cells – which exhibit periodic spatial firing fields that form a triangular lattice covering all environments visited by an animal (Figure 1B) [8] – were identified in the medial entorhinal cortex (mEC), a principal input to the hippocampus (Box 2). Finally, boundary vector/border cells (hereafter referred to as boundary cells) – which fire at a specific distance and direction from environmental boundaries (Figure 1C) – were identified in subiculum 9, 10, parasubiculum [11], and mEC 11, 12.

Box 1 **Other spatially modulated cell types of the hippocampal formation**

In addition to place and grid cells, the hippocampal formation contains several other spatially modulated cell types, including head direction cells 5, 6, 7, boundary cells 9, 10, 11, 12, and cells that encode object locations 99, 100.

Head direction cells, initially identified in the subiculum but subsequently throughout the Papez circuit, encode the animal's head direction in the horizontal plane, independent of location (Figure 4A) 5, 6. Head direction cells maintain their firing orientation in the dark, suggesting that they can be updated on the basis of self-motion [7]; and rotate coherently with grid and place cells when distal visual stimuli are moved, suggesting that they become coupled to sensory input with experience 53, 62.

Boundary cells of the subiculum 9, 10, parasubiculum [11], and mEC 11, 12 fire whenever a boundary is at a particular distance and direction from the current location of the animal, independent of head direction, and exhibit a second firing field at the same distance and direction to additional boundaries placed within a familiar environment (Figure 1C) 9, 10, 11. These cells also maintain their firing patterns in darkness and rotate with polarising visual stimuli, coherently with head direction and grid cells 10, 11.

Neurons in the lEC typically fire in response to non-spatial cues such as odour [101], but rarely show stable spatial tuning in an open field [102]. However, they can encode the relative distance and direction to the current or previous location of specific objects within an environment, and provide an equivalent level of spatial information to cells in mEC under these conditions (Figure 4B) 99, 100.

Box 2 **Anatomy of the hippocampal formation**

The hippocampal formation (HF) is composed of the dentate gyrus (DG) and cornu ammonis (CA) subfields, often referred to as the hippocampus proper; subiculum, pre- and parasubiculum; and the entorhinal cortex (EC), which is generally subdivided into medial and lateral subregions on the basis of cell morphology, connectivity patterns, and electrophysiological characteristics (Figure I) 60, 61, 102. Subcortical structures, including the medial septum, anterior thalamus, and mammillary bodies, project to all subfields of the HF via the fimbria–fornix fibre bundle 60, 61. In addition, medial and lateral EC receives neocortical input from postrhinal and perirhinal cortices, respectively, and send projections to the hippocampus via the perforant path, with layer II cells primarily terminating in DG and CA3 and layer III cells in CA1 and subiculum 60, 61. Pre- and parasubiculum are reciprocally connected, the former projecting primarily to layer III of the mEC and the latter projecting to layer II of both mEC and lEC, as well as the DG 60, 61, 104. Within the hippocampus, connectivity is traditionally characterised as a unidirectional polysynaptic loop consisting of mossy fibre projections from DG to CA3 and Schaffer collateral projections from CA3 to CA1 60, 61. CA1 sends output projections to the deeper layers of EC, both directly and via the subiculum; the subiculum also sends output projections to pre- and parasubiculum; and there are tentative reports of a back-projection from subiculum to CA1, although it is not clear if this is excitatory or inhibitory in nature 60, 61, 103, 104.

**Figure I:**
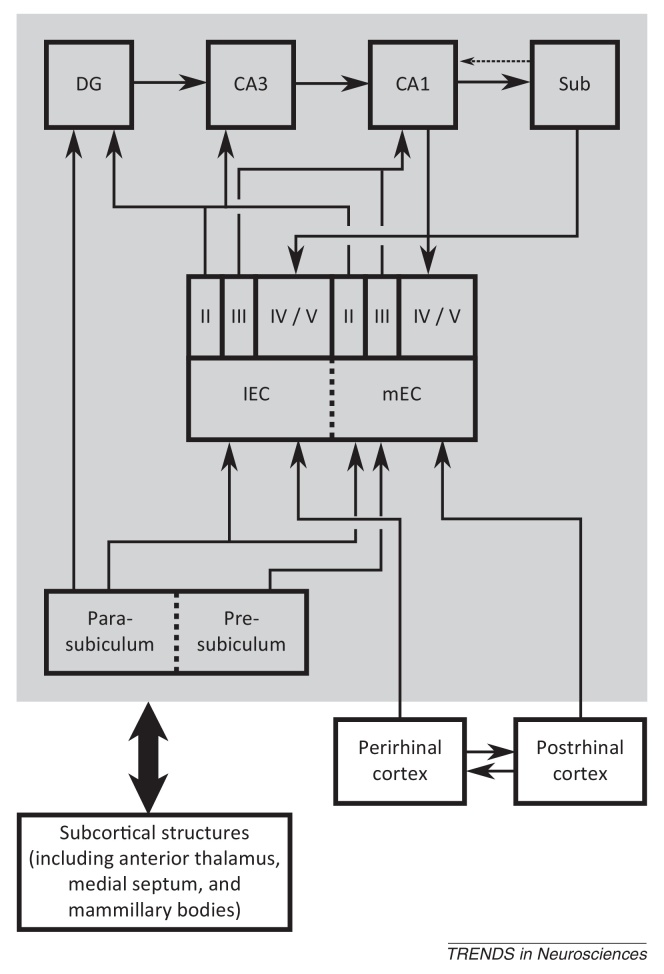
Anatomy of the hippocampal formation. Subcortical structures – including the medial septum, mammillary bodies, and anterior thalamus – project to all subfields of the hippocampal formation, most notably via the fimbria/fornix fibre bundle. Post- and perirhinal cortices provide neocortical input to medial entorhinal cortex (mEC) and lateral entorhinal cortex (lEC), respectively. The presubiculum projects to mEC, whereas the parasubiculum projects to mEC, lEC, and dentate gyrus (DG). Both mEC and lEC provide input to the DG, CA3, and CA1 subfields of the hippocampus proper via the perforant path. Within the hippocampus proper, DG sends mossy fibre projections to CA3, CA3 sends Schaffer collateral projections to CA1, and CA1 sends output projections to the deep layers of mEC and lEC both directly and via the subiculum.

Following the discovery of grid cells, several theoretical studies established that place fields could be generated by combining grid firing patterns with different spatial scales 13, 14, 15, 16, 17, 18, 19, 20, 21, 22, 23, 24, and grid cell input has subsequently come to be considered the primary determinant of place cell firing (e.g., [25]). However, recent studies have challenged this view by demonstrating that place field firing patterns are largely unaffected by an absence of stable grid cell activity. Here, we briefly review the properties of spatially responsive cells in the hippocampal formation, describe theoretical models of the grid cell to place cell transformation, evaluate the evidence for and against these models, and present an alternative view. In this view, place field firing patterns are primarily determined by environmental sensory inputs, including boundary cells (Box 3) 26, 27 to encode locations within specific spatial contexts, whereas grid cells provide a highly efficient and context-independent spatial metric for path integration and vector navigation. Thus, grid and place cells do not represent successive stages of a processing hierarchy, but rather provide complementary and interacting representations that work in combination to support the reliable coding of large-scale space.

Box 3 **The boundary vector cell model of place cell firing**

**Figure 2:**
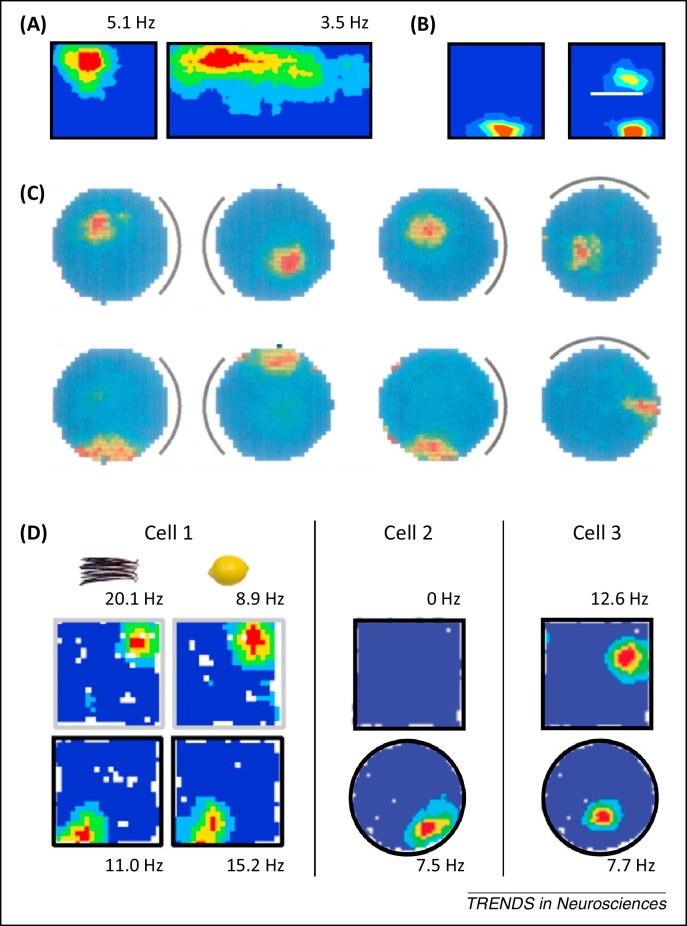
Factors controlling place field firing. **(A)** Place field deformation when a familiar environment is reshaped, illustrating a constant relationship between place cell firing and the allocentric distance to one or more environmental boundaries (adapted from [26]). **(B)** Appearance of a second place field when a novel boundary is placed in a familiar environment, illustrating how local boundaries control place cell firing locations (adapted from [9]). **(C)** Rotation of place fields with a distal visual cue (marked by a black line) in an otherwise symmetrical environment (reproduced, with permission, from [53]). **(D)** Rate and global remapping in place cells. Cell 1: a comparison of the left and right panels illustrates rate remapping, where place cell firing rates change with contextual cues (i.e., vanilla or lemon odour); whereas a comparison of the top and bottom panels illustrates global remapping, where place cells change their firing locations between different environments (i.e., black or white walls) (adapted from [57]). Cells 2,3: further examples of global remapping in place cell responses, where cells cease firing or change their firing locations between different environments (adapted from [59]).

The ‘boundary vector cell’ (BVC) model of place cell firing arose from the observation that place cell firing locations tend to maintain fixed distances to one or more boundaries following changes to the geometry of a familiar environment [26] (Figure 2A). These properties were hypothesised to reflect input from BVCs – putative cells that respond to the presence of an environmental boundary at a preferred distance and allocentric direction from the animal 27, 107 (Figure 1C]. Changes to place cell firing patterns following geometric manipulations of a familiar environment can then be predicted as a thresholded sum of a small number of BVC inputs (Figure I). For example, many place cells develop a secondary firing field with the same spatial relationship to a novel boundary that their initial firing field had to the original environmental boundaries (Figure 2B) 9, 27. Gradual changes to these firing patterns 35, 108 can also be explained by the action of an unsupervised learning rule on the synaptic connections from BVCs to place cells [109].

**Figure I:**
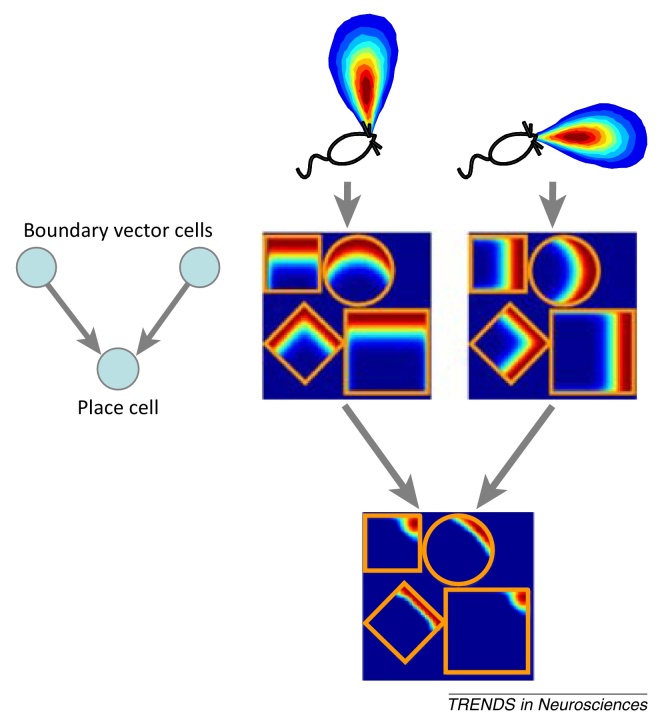
The boundary vector cell (BVC) model of place cell firing. Place cell firing patterns can be modelled as the thresholded sum of BVC inputs, which are tuned to respond to the presence of environmental boundaries at a fixed distance and allocentric direction from the animal. Putative firing rate maps are shown for two BVC inputs and a single place field output in four environments (adapted from [27]).

Several years after the development of this model, initial evidence for the existence of cells with the requisite spatial modulation was obtained from the subiculum [9], and the properties of these boundary vector or border cells (referred to here as boundary cells, for simplicity) have subsequently been more fully characterised in the medial entorhinal cortex 11, 12, parasubiculum [11], and subiculum [10]. Boundary cell receptive fields 10, 11, similarly to those of place [53] and grid cells [62], rotate coherently with head direction cell tuning curves, suggesting that the latter provide global orientation input for each of these spatially selective cell types [10]. Moreover, boundary cells continue to fire at a fixed distance and allocentric direction from boundaries across very different environments, while simultaneously recorded place cells exhibit global remapping [10], suggesting that additional contextual inputs must also influence place cell firing patterns.

## Place cells

Place cells, most often studied in rats, are typically complex spiking pyramidal cells of the CA3 and CA1 hippocampal subfields 4, 28. CA1 and CA3 place cells generally exhibit a single place field, but sometimes several in larger environments 28, 29. In addition, granule cells in the dentate gyrus (DG) can exhibit several, smaller place fields [30]. Place fields are established rapidly in a novel environment 31, 32, 33 and remain stable between visits to an environment [34] while slowly evolving over longer timescales 35, 36. Place cells are present throughout the dorso-ventral axis of the hippocampus, but place fields are larger towards the ventral pole 37, 38, 39. Place cell activity is typically observed during translational movement, which is associated with 5–10 Hz theta oscillations in the local field potential (LFP) [40]. During these periods, place cells exhibit theta phase precession – that is, their firing phase relative to theta is negatively correlated with the distance travelled through the place field 41, 42.

What factors are known to modulate place cell firing? First, evidence suggests that place fields are controlled by local boundaries, as firing often occurs at fixed distances from boundaries in one or more allocentric directions across geometrically deformed versions of an environment (Figure 2A) 26, 27, 43, 44, and secondary firing fields often develop in the same position relative to a new boundary placed into the environment (Figure 2B) 27, 35. Second, it is believed that place cells receive inputs reflecting self-motion 44, 45, 46, 47, 48, 49. For example, when environmental and self-motion cues are put in conflict, firing field locations of a significant proportion of place cells are specifically influenced by movement related information 26, 43, 44, 45. Third, place cell responses are oriented to distal visual cues. For example, if a polarising visual cue in a circular environment is rotated, then the positions of place fields within that environment rotate correspondingly (Figure 2C) 50, 51, 52, coherent with head direction cell responses [53]. Proximal sensory cues can also exert some control over place cell firing 50, 53, 54. Finally, not all place cells are active in all environments. Although approximately 90% of principal cells in the dorsal hippocampus can exhibit place fields, only 15–50% do so in any given environment 32, 43, 55, and there appears to be no relationship between the subset of cells that are active in different environments and the location of their firing fields 50, 51, 55. Minor manipulations of environmental features may modulate the firing rate of active place cells, particularly in CA3 (‘rate remapping’), whereas larger manipulations of the environment can change the entire ensemble of active place cells and their firing locations (‘global remapping’; Figure 2D) 56, 57, 58, 59.

## Grid cells

**Figure 3:**
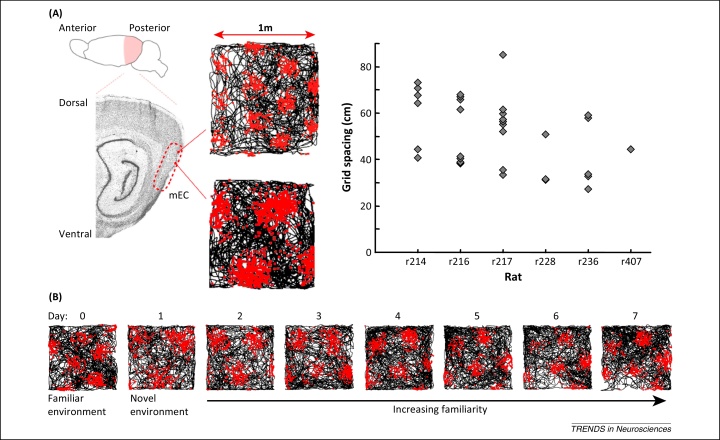
Properties of grid cell firing. **(A)** The spatial scale of grid firing fields increases in discrete steps along the dorso-ventral axis of the medial entorhinal cortex (mEC). Spike rasters for two grid cells recorded from the same animal at different positions along the dorso-ventral axis of mEC are shown alongside the scale of all grid cells recorded in six rats, illustrating the discrete nature of grid scale increases (adapted from [65]). **(B)** Grid cell firing patterns expand in novel environments. Spike rasters for a single grid cell recorded over several days illustrate that firing field size and spatial scale increase upon exposure to a novel environment and then progressively decrease with experience until they return to their original scale, observed in familiar environments (adapted from [68]).

A principal neocortical input to the hippocampus arises in the superficial layers of mEC 60, 61, where grid cells are the most numerous spatially modulated cell type 8, 62. Grid cells exhibit periodic spatial receptive fields that form a remarkably regular triangular lattice covering all environments visited by the animal [8]. Grid cells have also been identified in the deeper layers of mEC, where their firing rates are often modulated by head direction [62], and in pre- and parasubiculum [63]. Grid cells in the superficial layers of mEC exhibit theta phase precession that is independent of the hippocampus [64]. Like place cells, the scale of the grid firing pattern increases along the dorso-ventral axis of mEC [8], but this increase occurs in discrete steps, with grid cells at each discrete scale appearing to exist in independent modules (Figure 3A) 65, 66. The scale, relative orientation, and offset of grid firing patterns within each module are generally conserved across environments [67], aside from temporary expansion when encountering a novel environment (Figure 3B) [68], and their firing patterns are maintained in the dark [8]. This has led to the suggestion that grid cells perform path integration, updating their firing patterns on the basis of self-motion [69]. However, grid firing patterns also remain stable between visits to an environment 8, 67, are oriented by distal cues [8], and parametrically rescale when a familiar environment is deformed [65], suggesting that they become attached to environmental sensory information with experience. Finally, the relative spatial phase and orientation of grid cell modules can shift between environments, concomitant with global remapping in place cells [67].

**Figure 4:**
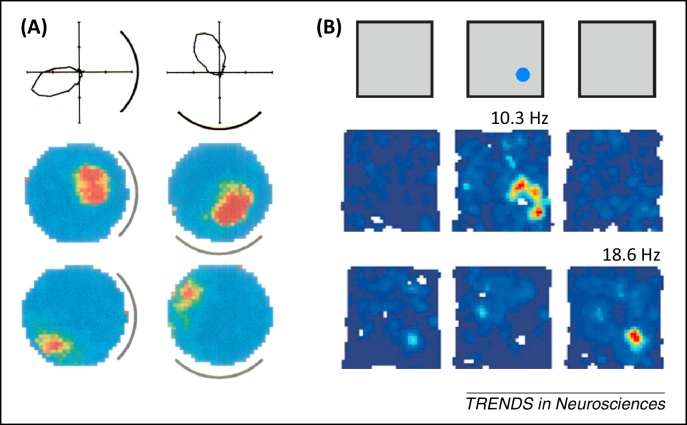
Other spatially receptive cell types of the hippocampal formation. **(A)** Head direction cells of the anterior thalamus. Head direction cell firing is not spatially modulated but strongly dependent on the animal's head direction in the horizontal plane. Top row: polar plots illustrate the directionally tuned activity of a single head direction cell recorded in a familiar environment, and how the preferred direction of that cell rotates with a distal visual cue (marked by a black line). Bottom rows: simultaneously recorded place cell responses illustrate how place field firing locations rotate coherently with the preferred orientation of head direction cells (reproduced, with permission, from [53]). **(B)** Object and object trace cells of the lateral entorhinal cortex. Top row: three recording sessions were performed in a familiar environment, with an object (marked by the blue dot) placed into the environment for the second session but removed for the third. Middle row: firing rate map of a typical object cell, which encodes the location of the object when it is present within the environment. Bottom row: firing rate map of a typical object trace cell, which encodes the previous position of objects that are no longer present within the environment (adapted, with permission, from [100]).

## Grid cell to place cell models

Following the discovery of grid cells, multiple theoretical models demonstrated how input from grid modules of two or more spatial scales could be combined to generate place fields through an effective Fourier synthesis 13, 14, 15, 16, 17, 18, 19, 20, 21, 22, 23, 24. These models use hardwired synaptic weights 16, 17, a heterosynaptic Hebbian learning rule 15, 18, 20, 21, 22, and/or competitive network interactions 14, 15, 18, 19, 22, 23, 24 to set the effective strength of grid cell inputs to decline with their spatial offset from the output place field [70]. Assuming that activity in the grid cell network is driven by movement related input [69], these models can then account for the update of place cell firing by self-motion. Grid cell to place cell models can produce either single or multiple place fields, although the secondary fields often exhibit six fold symmetry, in contrast to empirical data 14, 16, 22, 24. This issue is particularly common when all grid inputs share a single orientation [16], and it is known that the orientation of grid modules within a single animal tend to be clustered 65, 66. However, more restricted place field firing can be generated by introducing some variation in firing rate between the receptive fields of each grid cell 15, 24, in line with experimental data [8]. Finally, making independent changes to the orientation and/or spatial phase of input from each grid module 14, 23, or incorporating a ‘gating’ input representing abstract contextual signals 17, 24, can account for both rate and global remapping of output place field responses.

## Evidence supporting grid cell to place cell models

Several strands of empirical evidence have been offered in favour of the hypothesis that grid firing patterns are the main determinant of place cell firing. First, grid cells are the most numerous spatially modulated cell type in the superficial layers of entorhinal cortex 8, 62, the principal neocortical input to the hippocampus 60, 61. Recent combined optogenetic–electrophysiology experiments confirmed that a significant proportion of excitatory projections to place cells that arise in mEC come from grid cells, along with several other spatial and non-spatial cell types [71]. Place fields become less spatial towards proximal CA1, where mEC inputs are fewer [72], and both grid 65, 66 and place fields 37, 38, 39 are larger towards the ventral pole, consistent with grid cell to place cell models that incorporate topographic projections along the dorso-ventral axis 16, 24. Moreover, dorsal hippocampal place fields decrease in size after lesions of ventral and intermediate EC [73], consistent with the proposed convergence of input from grid cells covering a range of scales 16, 24, although conflicting results have been reported [74].

Second, evidence in favour of a functional projection from grid cells to place cells during navigation comes from the suggestion that place cell phase precession relies on extra-hippocampal mechanisms 75, 76. Silencing CA1 pyramidal cells and interneurons for one or more theta cycles while the animal continues to navigate freely does not prevent place cells discharging at the appropriate theta phase when firing activity resumes, consistent with hippocampal phase precession arising from external inputs [75]. Moreover, bilateral mEC lesions have been shown to abolish phase precession in CA1 place cells without eliminating spatially selective firing [76]. The fact that grid cells represent the only known cell type projecting to the hippocampus which exhibit theta phase coding [64] makes them most likely to account for the place cell temporal code. However, non-spatially modulated cells whose theta firing frequency is modulated by movement velocity have also been identified in the septo-hippocampal circuit [77], and these would be sufficient to produce both phase precession [78] and arbitrary spatial firing patterns [77] in target neurons.

Third, removing input from CA3 does not eliminate CA1 place cell responses in novel or familiar environments 79, 80, although firing field size is increased and spatial information content reduced [80], suggesting that place cell firing can be established and maintained by direct entorhinal input. However, removing inputs from mEC to CA1 also fails to eliminate place cell responses in novel or familiar environments, although it does cause a reduction in the frequency of pyramidal cells exhibiting place fields 25, 81, an increase in firing field size 25, 74, 76, and a reduction in spatial information content ([76], but see [73]). Similarly, removing input from subcortical structures [81] or pre- and parasubiculum [82] reduces the frequency and spatial information content of place fields in familiar environments, but does not eliminate place cell responses. Hence, it appears that input from CA3, mEC, subcortical structures, pre- and parasubiculum all contribute to the generation of sharp CA1 place fields.

Finally, in accordance with theoretical models 14, 23, experimental studies have demonstrated that global remapping of place cell firing is accompanied by shifts in the spatial phase and/or orientation of grid cell modules relative to the environment [67]. However, this relationship is correlative rather than causal, and does not indicate whether grid module shifts drive changes in place field firing or vice versa. Furthermore, rate remapping of place cell responses is not associated with changes in grid cell firing rates or grid field shifts [67], but is compromised by lesion of the lateral entorhinal cortex (lEC) [83], consistent with models which suggest that contextual input from lEC gates spatially modulated input from mEC to modulate place cell firing rates 17, 24. In accordance with this hypothesis, recent intracellular recordings *in vivo* demonstrate that place field responses can be unmasked by tonic depolarisation of a principal cell that previously exhibited no spatially modulated subthreshold membrane potential fluctuations during navigation, possibly mimicking the effects of contextual input from lEC [84].

## Evidence against grid cell to place cell models

Further recent research has challenged the view that grid cell responses give rise to place cell firing by demonstrating that hippocampal place fields are largely unaffected by an absence of effective input from the grid cell network. First, significant proportions of stable, adult-like place fields are present when pre-weanling rats first leave the nest and actively navigate, whereas significant proportions of stable grid firing patterns do not appear until several days later, suggesting that place cell responses are initially established in the absence of grid like firing 85, 86. Although some mEC cells do exhibit spatially selective firing earlier in the developmental timeline, their firing fields lack sufficient inter-trial stability to account for the stable place fields that are observed ([86], but see [85]). Interestingly, adult-like head direction cell activity is present from the very first excursion outside of the nest 85, 86, and adult-like boundary cell activity develops much earlier than grid cell responses 87, 88.

Second, both grid scale and grid firing field size increase significantly upon exposure to a novel environment, and grid firing patterns remain expanded for several hours as the environment becomes familiar [68]. Conversely, place field location rapidly becomes stable in a novel environment 31, 32, 33, and the temporary increase in place field size returns to baseline with a much faster time course [68]. This suggests that spatially modulated input from grid cells continues to change long after stable place cell responses have been established.

Third, inactivation of the medial septum reduces theta rhythmicity and eliminates the spatial periodicity of grid cell firing with little effect on the maintenance of place fields in familiar environments [89] or the formation of place fields in novel environments [90], despite a significant reduction in place cell firing rates [89]. Although medial septum inactivation does not completely disrupt grid cell spatial selectivity, and the inter-trial stability of place fields is significantly reduced, place field locations are significantly better preserved than those of the remaining grid cell firing fields, suggesting that they cannot be wholly accounted for by grid cell inputs (Box 4) [89]. Interestingly, this reduction in theta rhythmicity has little effect on head direction cells, the directional component of conjunctive cells, or the firing patterns of boundary cells 89, 91.

Box 4 **Outstanding questions**

• Can analysis of the relative spike timing in different spatially modulated cell types during movement related theta elucidate the causal relationships between boundary, grid, and place cell firing [92]? Do these temporal relationships change according to behavioural requirements – for example, might grid cell input to place cells be more important when environmental sensory cues are reduced [118], or differ between novel and familiar environments?
• What is the relationship between grid and boundary cell firing in mEC 8, 11, 12, 62, subiculum 9, 10, and pre- and parasubiculum 11, 63? Do boundary cells stabilise grid cell firing patterns [11], or do they only interact via place cells [110]? Are grid and boundary cell firing patterns in some regions inherited from elsewhere, or do they arise independently in each region?
• What explains the stable differences in firing rate between the spatial receptive fields of a grid cell [8]? If the grid cell network represents a context-independent spatial metric 94, 106, then there is no clear role for this firing rate heterogeneity. However, if grid cell in-field firing rates are controlled by environmental sensory cues, they may contribute to variations in place cell firing that encode local attributes of space [42]. Alternatively, they might represent a deviation from ‘pure’ grid like firing that reflects ‘imperfect’ combinations of underlying periodic inputs 93, 111, 112.
• What inputs are necessary for the global remapping of place cell responses? It has been shown that lEC input makes some contribution to remapping [83], but are the expansion [68], shift, and/or rotation [67] of grid firing patterns in a novel environment also necessary? This could be explored using selective lesions, inactivation of the medial septum to eliminate grid cell responses without affecting place cell firing patterns, or optogenetic techniques.
• What are the relative contributions of environmental and self-motion information to place cell firing? Do place cells form a pre-configured chart driven by self-motion (via grid cells [69]) which then becomes associated to sensory input in a particular environment [113]? Or are place fields initially determined by sensory inputs (via boundary cells [27]) and then become associated to grid cells as an environment becomes familiar 68, 110? Or are place fields near to environmental boundaries driven by boundary cells, and those far from the boundary more reliant on grid cells [88]?
• Which aspects of medial septal inactivation are responsible for the observed effects on place and grid cell firing? The oscillatory interference model 13, 77, 78, 93 suggests that the loss of grid cell spatial periodicity and stability reflects the disruption of theta rhythmicity, consistent with the correlation between these variables 89, 91. By contrast, the reduction of place field stability [89], especially in novel environments [90], resembles the effects of impairing synaptic plasticity 114, 115, suggesting that they reflect the disruption of cholinergic input to the hippocampal formation, which impairs synaptic plasticity 116, 117. These possibilities could be dissociated by optogenetic or pharmacological manipulations that specifically target cholinergic neurons or inhibitory theta cells in the medial septum.

Finally, it has been demonstrated that both principal cell and interneuron activity in mEC peaks shortly after principal cell activity in the hippocampus during theta-associated behaviour, making a causal contribution unlikely [92]. However, it is important to note that this analysis did not distinguish between cells in mEC or hippocampus on the basis of their spatial firing patterns. Furthermore, the firing probability of principal cells in mEC layer II with an instantaneous rate of ≥40 Hz, which may be most effective in driving target neurons, does peak shortly before that in their hippocampal afferents [92].

## An alternative model of place cell firing

The evidence discussed above indicates that place fields can be both established and maintained in the absence of stable input from the grid cell network [118]. What then can account for the formation of highly selective, spatially stable place field firing, and what contribution might be made by grid cells?

We suggest that place cell firing is primarily driven by environmental sensory inputs from boundary cells 9, 10, 26, 27, 35 in mEC (Box 3) 11, 12, 89. This hypothesis is supported by several aspects of the empirical data. First, the position of place fields in altered environments can be strongly predicted by their position relative to previous boundaries 26, 27, 35, and additional place fields often develop in the same relative position to an additional boundary placed in a familiar environment 26, 27, consistent with input from boundary cells 9, 10. Second, although boundary cells constitute a smaller proportion of mEC principal neurons than grid cells, they appear to be at least as likely to project to principal neurons in the hippocampus [71]. Third, boundary cells appear earlier in the developmental timeline, making a causal contribution to stable place cell responses more likely 87, 88. Fourth, their firing patterns are rapidly expressed and stable in novel environments, in which grids gradually contract 10, 68. Finally, they are not affected by a reduction in theta rhythmicity, potentially accounting for the persistence of place field responses following inactivation of the medial septum 89, 91.

This hypothesis does not preclude a contribution of grid cell activity to place cell firing patterns, however. Grid cells are the most common spatially modulated cell type in mEC 8, 62, which is the most significant neocortical input to the hippocampus 60, 61, and therefore highly likely to influence place cell responses. Existing data suggest that self-motion information provided by grid cells 13, 69 could help to maintain the spatial stability of place cell firing 89, 90, although direct evidence is so far lacking. The limited evidence for boundary cells that fire at a distance from environmental borders 10, 11, 12 might suggest that grid cell inputs are particularly important for maintaining place fields towards the centre of an open environment. This would be consistent with developmental data [88] and with the greater influence of proximal versus distal boundaries on place cell firing fields 26, 43, 52. However, the distribution of preferred response distances for mEC boundary cells has not yet been characterised, and several examples of cells that respond at larger distances have also been reported (see ‘spatial non-grid cells’ in the supporting online material of [89]).

It also seems likely that the theta phase precession of place cell firing is inherited from grid cell inputs (75, 76, but see [77]), suggesting that temporal coding in the grid cell population might be associated with path integration mechanisms, consistent with several theoretical models 13, 77, 78, 93. Moreover, grid firing patterns represent a constant spatial metric that could, in principle, allow a translational vector between locations to be extracted and used to support novel shortcutting over large distances 94, 95. This is not true of place cell firing patterns, which can directly support navigation over distances up to the scale of the largest place fields [96], but require an additional, potentially slow, learning mechanism over larger distances [97]. Finally, grid scale expansion in novel environments [68], along with the rotation and shift of grid firing patterns between familiar environments [67], may help to drive global remapping.

The data discussed above also indicate that input from other spatially receptive cells in the hippocampal formation likely contributes to place cell firing. First, the influence of proximal sensory cues 52, 54, 98 could reflect input from spatially modulated lEC neurons 99, 100. Furthermore, non-spatial or ‘contextual’ inputs from lEC 101, 102 could modulate boundary cell firing to account for place cell remapping in geometrically similar environments 35, 56, 57, 58, 59, consistent with lesion data [83] and theoretical models (Box 4) 17, 24. Second, the coherent rotation of place, grid, and boundary cell receptive fields with those of simultaneously recorded head direction cells might indicate that the latter provide global orientation information for all spatially receptive neurons in the hippocampal formation 10, 11, 53, 62, 98.

Some empirical data still present a challenge to the hypothesis that boundary and grid cells make a causal contribution to generating place field firing patterns, however. First, the theta phase of peak activity in the place cell population appears to precede that in mEC principal neurons, which include boundary and grid cells, although the former are less numerous and therefore make a smaller contribution to the pooled spike timing data (Box 4) [92]. Second, place fields can be established and maintained following mEC lesion, which presumably eliminates the majority of both boundary and grid cell inputs 25, 73, 76, 81. In these circumstances, inputs from boundary and/or grid cells in pre- and parasubiculum to DG [63], from boundary cells in subiculum to CA1 9, 10, 103, 104, and from lEC 99, 100 and subcortical structures [77] to all hippocampal subfields, are presumably sufficient to support place cell firing (Box 4).

Finally, it is important to emphasise that the hippocampal formation represents a processing loop in which CA1 place cells provide significant return projections to grid cells in the deeper layers of mEC, pre-, and parasubiculum 60, 61, 62, 63. Grid firing patterns are oriented by polarising visual cues, stable between visits to an environment, and parametrically rescaled when a familiar environment is reshaped, demonstrating that grid cells receive environmental sensory input that may be provided by place cells 8, 65. This is consistent with the firing of mEC principal cells following that in hippocampus during movement related theta [92], and the observation that inactivation of the hippocampus eliminates grid firing patterns [105]. Place cell input may serve to reduce accumulating path integration error in the grid cell network, consistent with the fact that grid firing patterns are less coherent in novel environments, before associations with sensory information may have developed [68].

In this view, the grid and place cell networks provide complementary spatial representations that interact to support accurate navigation and mnemonic function: grid cells constitute a highly efficient, context-independent spatial code that supports path integration and large-scale vector navigation 94, 95, 106, whereas place cells integrate multimodal sensory information to encode defining cues at specific locations in support of episodic memory. Interaction between these networks is crucial for accurate navigation across large-scale space – connections between place cells and grid cells could associate specific environmental locations with their corresponding ‘grid coordinates’ in support of vector navigation; and could also provide a powerful error correction mechanism for path integration because small errors in grid field firing will correspond to locations outside the current environment [106]. Although this view sees place cells as essential for encoding the conjunction of sensory stimuli at a specific location, which may underpin their putative role in episodic memory, it is less clear if the path integrative and strongly spatial correlates of grid cells also contribute to episodic memory function.
